# Is there evidence of a causal link between childhood maltreatment and attention deficit/hyperactivity disorder? A systematic review of prospective longitudinal studies using the Bradford‐Hill criteria

**DOI:** 10.1002/jcv2.12169

**Published:** 2023-05-27

**Authors:** Paraskevi Bali, Edmund Sonuga‐Barke, Christina Mohr‐Jensen, Ditte Demontis, Helen Minnis

**Affiliations:** ^1^ University of Glasgow Institute of Health and Wellbeing Glasgow UK; ^2^ Department of Child and Adolescent Psychiatry Institute of Psychiatry, Psychology & Neuroscience King's College London London UK; ^3^ Department of Child and Adolescent Psychiatry Aalborg Psychiatric Hospital Aalborg University Hospital Aalborg Denmark; ^4^ Department of Biomedicine ‐ Human Genetics Aarhus University Aarhus Denmark; ^5^ The Lundbeck Foundation Initiative for Integrative Psychiatric Research iPSYCH Aarhus Denmark

**Keywords:** ADHD, causality, childhood maltreatment

## Abstract

**Background:**

Studies report an elevated risk of maltreatment in children with attention deficit/hyperactivity disorder (ADHD), and elevated levels of ADHD in people who suffered childhood maltreatment (CM). However, the direction(s) of causality between CM and ADHD remain unclear—does ADHD create a context for CM, does CM cause ADHD, or both?

**Objective:**

This study systematically reviews and qualitatively synthesizes the research evidence relating to this question using Bradford‐Hill criteria for establishing causality—*strength, temporality, dose‐response* and *plausibility.*

**Methods:**

We conducted a systematic review, following PRISMA guidelines, of prospective longitudinal studies examining both CM and ADHD. We then used Bradford‐Hill criteria to assess the quality of evidence for a causal link between CM and ADHD.

**Results:**

All 11 included studies demonstrated an association between CM and ADHD. Seven included evidence for temporality: five suggesting that CM precedes ADHD in the lifespan; two suggesting ADHD precedes CM. Four studies demonstrated a dose response relationship in which greater CM exposure was associated with elevated risk of ADHD. Studies presented a range of plausible mechanisms, including CM causing ADHD through biological programming, versus ADHD causing CM through parental stress.

**Conclusions:**

The high quality prospective longitudinal studies reviewed confirm the association between ADHD and CM, but present conflicting evidence about the direction of causality and mechanisms underpinning this association. To better understand the complex interplay between CM and ADHD, more studies using new research designs will be required that can partition effects by type of CM and account for bidirectional effects and other complexities.

**Key points**

- There is an association between attention deficit/hyperactivity disorder (ADHD) and childhood maltreatment (CM), irrespective of which condition precedes the other.
- There is no consistency in the included studies about the direction of causality—probably because they involve CM exposure that differ in type and degree.
- There is dose‐response relationship: multiple or more frequent exposure to CM is associated with higher risk of ADHD symptoms.
- Although authors suggest various plausible explanations for the association between CM and ADHD, further research is needed to disentangle the complexity of this association.

## INTRODUCTION

Childhood maltreatment (CM), defined as physical abuse and neglect (including malnutrition) emotional abuse and neglect; sexual abuse; and early deprivation (APA, 2013; WHO, 2004), is associated with negative health outcomes in both the short‐ and long‐term (Baldwin et al., 2023; Kerr et al., 2021; Lang et al., 2020). The association between CM and attention‐deficit/hyperactivity disorder (ADHD) is well established, but its origins and nature are still uncertain (Briscoe‐Smith & Hinshaw, 2006; Capusan et al., 2016; Sanderud et al., 2016; Wang et al., 2020). There are several hypotheses in the literature regarding the association between CM and ADHD. Two common hypotheses are:

Hypothesis A CM is a causal factor in the etiology of ADHD through biological programming in which adverse experiences are embedded (Danese & McEwen, 2012). For example, CM can activate the Hypothalamus‐Pituitary‐Adrenal Axis, with acute and long‐term effects on brain functioning and development (Ioannidis et al., 2020; Mehta et al., 2013), altering brain structures (e.g. amygdala, hippocampus, and the prefrontal cortex) (Kessler et al., 2018), leading to impairments in proactive inhibition, memory, decision‐making and emotion recognition—all implicated in ADHD (Golm et al., 2020; Sonuga‐Barke et al., 2017). Also, the McLaughlin and Sheridan model (2021) suggests that childhood adversity (including CM) disrupts learning processes (emotional, associative, fear and reward) with consequent developmental outcomes, including psychopathology (ADHD).

Hypothesis B Symptoms of ADHD in children, such as externalizing problems, may evoke negative responses from caregivers, and peers (Boyd et al., 2019; Capusan et al., 2016; Ouyang et al., 2008; Sugaya et al., 2012) and this could lead to CM. ADHD often runs in families. GWAS studies have found that individuals with high ADHD‐PRS, have greater risk of experiencing maltreatment (He & Li, 2022; Ratanatharathorn et al., 2021). Parental ADHD symptoms such as impulsivity, attention problems and executive dysfunction could interfere with the parent's ability to maintain a safe and predictable environment for their children, increasing risk‐factors for CM, and the tendency to develop ADHD could be inherited by the child (Fujiwara et al., 2014; Fuller‐Thomson et al., 2014).

*Inferring cause from observational data—the Bradford‐Hill criteria*:

For more than 50 years the Bradford Hill criteria have been used as a basis for establishing causal inference from observational data about the association between an environmental exposure (putative cause) and a disease outcome (Hill, 1965).

Four core Bradford‐Hill criteria can be applied to an individual study (Fedak et al., 2015): *strength, dose‐response*, *plausibility and temporality.* The *stronger* the association is, the more likely that the association is to be causal. A *dose‐response* relationship between an exposure and an outcome means that the more frequent (or more severe) the exposure, the greater the risk of the outcome. *Temporality* refers to the necessity of a temporal relationship between exposure and outcome. Epidemiologists agree that temporality offers crucial information about the direction of causality. Prospective longitudinal studies are therefore regarded as essential in teasing apart direction(s) of causality as they avoid the potential biases inherent in retrospective assessments of CM and ADHD (Baldwin et al., 2019; Danese & Widom, 2020). Finally, a biologically *plausible* explanation supports the drawing of a causal conclusion.

A further five Bradford Hill criteria can be used to examine a body of literature as a whole and we critically examine these in the Discussion section of the paper, across all of the studies in the review. These are *coherence* (between current findings and the previous literature)*, consistency* (across the studies), *specificity* (is the causal component sufficient to produce the outcome?)*, experimental evidence* (does altering the exposure in a randomized controlled trial alter the outcome?), and *analogy* (are there analogous mechanisms in other fields?).

### Aims of the current review

We systematically reviewed prospective longitudinal studies (published between 1985 and 2023) that included measurement of CM and/or ADHD at a minimum of two time points. Exclusion of studies prior to 1985 was to allow use of modern definitions of ADHD in accordance with the publication of DSM‐III. We assessed the extent to which findings of each study satisfied Bradford Hill criteria, asking:

1. What is the *strength of the association between CM and ADHD?*
2. Did CM precede ADHD or vice versa?
3. Was there evidence for a dose response relationship between either the intensity or duration of the CM exposure and ADHD?
4. How plausible is the effect?

## METHODS

The systematic literature review was conducted following the PRISMA guideline (Page et al., 2021).

PubMed, Scopus, ScienceDirect, PsycINFO, and Cochrane library were searched using the following terms: Childhood maltreatment, Childhood ADHD, attention deficit/hyperactivity disorder, deprivation, maltreatment, (ADHD) AND (maltreatment), (ADHD) AND (childhood maltreatment), (ADHD) AND (maltreatment) AND (children), ((ADHD) AND (Childhood maltreatment)) AND (longitudinal studies), ((Sexual Abuse) AND (ADHD)), ((Physical Abuse) AND (ADHD)), ((emotional Abuse) AND (ADHD)), (Physical neglect) AND (ADHD), (emotional neglect) AND (ADHD), (attention deficit) AND (maltreatment), (attention deficit) AND (maltreatment), (hyperactivity) AND (maltreatment), (deprivation) AND (ADHD), (deprivation) AND (attention deficit), (deprivation) AND (hyperactivity).

The initial search was carried out in June 2020 and updated in March 2022 and January 2023. We excluded abstracts, conference proceedings, reviews, meta‐analyses, retrospective studies and RCTs (if the trial did not include post‐trial follow‐up); and studies without adequate assessment of symptoms or diagnosis conducted by professionals using standardized clinical methods. After removing duplicates, all remaining articles were checked in a first (title, abstract, key words) and second (whole article) screening. The initial PRISMA search was performed independently by two reviewers. Full papers were reviewed by three co‐authors to decide which articles were selected for the final review.

### Eligibility criteria

Articles that were available in English and were published between years 1985–2023 were selected based on the following inclusion criteria:

- The study was prospective and longitudinal.
- The population of interest was children (including adolescence) followed for a minimum of 1 year.
- Eligible studies needed to have measured CM and/or ADHD at a minimum of two timepoints including at least one assessment of CM and at least one assessment ADHD before age 18 years.
- Studies included ADHD diagnosis and/or symptoms as exposure or outcome, and one or more forms of CM: physical neglect and abuse, sexual abuse, emotional (psychological) neglect and abuse, or early deprivation.
- Where a cohort had more than one publication associated with it, we selected the most recent publication that focused specifically on ADHD and CM.
- Studies were judged by two independent raters to be “high quality” see below under risk of bias assessment.

### Study risk of bias assessment

To assess the quality of the included studies and the risk of bias on estimates, we used the Crowe Critical Appraisal Tool (CCAT, version 1.4) (Crowe, 2013). CCAT creates a score out of 5 for each of the following domains: *preliminaries, introduction, design, sampling, data collection, ethical matters, results,* and *discussion*, resulting in a total score out of 40. A score of <20 is considered low quality; 20−30 moderate quality, and >30 high quality. Quality assessment for each included article was completed independently by two reviewers we included only high‐quality studies.

Evidence for causality in the association between CM and ADHD was assessed using Hill (1965) guidelines for causation (Howick et al., 2009). We applied the Cohen's *d* formula to examine the effect size of the results in each study (Cohen, 1962).

## RESULTS

### Study selection

**FIGURE 1:**
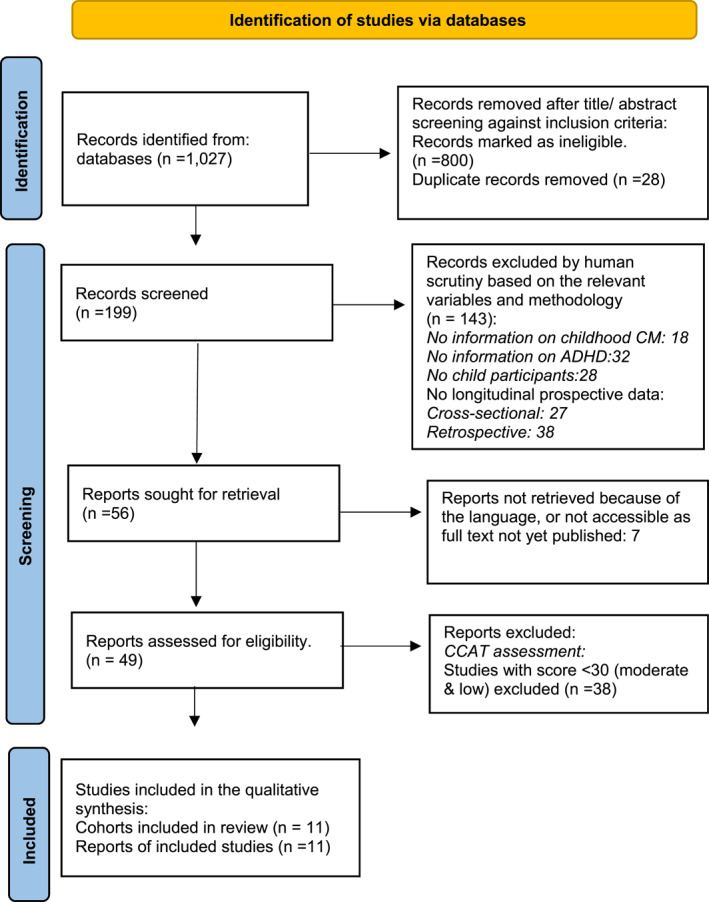
Flow diagram for study search and selection (PRISMA, 2020).

Study selection is presented in the PRISMA diagram Figure 1. A total of 1027 studies were identified using the search terms. Eleven papers describing prospective longitudinal studies based on 11 cohorts were included: Wong et al. (2022), Boyd et al. (2019), González et al. (2019), Calhoun et al. (2019), Stern et al. (2018), Hunt et al. (2017), Guendelman et al. (2016), Kennedy et al. (2016), Galler et al. (2012), Young et al. (2011), and McLaughlin et al. (2010). A list of excluded studies is available on request.

### Study characteristics

A total of 56,019 children and 14,770 adults with childhood CM and ADHD (including controls) from 11 prospective longitudinal studies were included. The participants were all children at the time of study initiation in 10 studies (Calhoun et al., 2019; González et al., 2019; Guendelman et al., 2016; Hunt et al., 2017; Kennedy et al., 2016; McLaughlin et al., 2010; Stern et al., 2018; Wong et al., 2022; Young et al., 2011) and two studies recruited both children and parents (Boyd et al., 2019; Galler et al., 2012) (see Table S1). Six studies followed the participants from childhood into adulthood (Boyd et al., 2019; Galler et al., 2012; Guendelman et al., 2016; Kennedy et al., 2016; Stern et al., 2018). Six studies conducted follow‐ups during childhood and adolescence (Calhoun et al., 2019; González et al., 2019; Hunt et al., 2017; McLaughlin et al., 2010; Wong et al., 2022; Young et al., 2011). Many of the studies mentioned forms of adversity that occurred beyond childhood, and we do not discuss these additional forms of adversity (e.g., domestic violence) in this paper. When articles were based on the same sample, we used the most relevant and most recent. For example, Humphreys et al. (2020) and Troller‐Renfree et al. (2018) are based on Bucharest Early Intervention Project (BEIP) sample and Lugo‐Candelas et al. (2021) uses the same sample as González et al. (2019) study. These three studies are more recent papers but have a broader focus than simply on ADHD and CM.

#### Early deprivation

In two studies CM was primarily linked to institutional deprivation (Kennedy et al., 2016; McLaughlin et al., 2010). Both have very high quality CCAT scores (36 and 38).

The English‐Romanian Adoption Study (ERA) (Kennedy et al., 2016) takes advantage of a natural experiment created when children experienced varying durations of severe deprivation within Romanian institutions (from 1 to 43 months) before being adopted into UK families in the late 1980s. The original sample was 165 Romanian Adoptees and 52 comparison UK adoptees. 164 young adults (76%) were followed up. ERA examined the association between early deprivation and neurodevelopmental conditions longitudinally and used multiple research methods to test both environmental and genetic risk.

The McLaughlin et al. (2010) study is based on the BEIP, a randomized controlled trial (RCT) focusing on a sample of 117 Romanian children raised in institutions, in which young children were randomly allocated to either stay in an institution or be placed in foster care, plus a Romanian family‐reared community control group. McLaughlin and colleagues used BEIP as a prospective longitudinal cohort study since findings were not analyzed according to group (institutionalized vs. foster care). Instead, the entire cohort, plus the never‐institutionalized Romanian comparison group, was followed up at 54 months examining brain development using electroencephalogram (McLaughlin et al., 2010).

#### Malnutrition

Galler et al. (2012) focused on the long‐term effects of infantile malnutrition by prospectively following up 80 malnourished infants into adulthood and comparing them, on assessments of attentional problems, with 65 healthy comparison participants in adulthood. Although malnutrition is often not a form of maltreatment but, instead, a feature of social circumstances, we have included it here because it can occur as part of extreme physical neglect.

#### Emotional neglect

Young et al. (2011) is a school based longitudinal study exploring the associations between children's perceptions of parental emotional neglect and control, and their future psychopathology.

#### Multiple forms of CM

Seven of the studies examined the association between multiple forms of maltreatment and ADHD. Three of these (Boyd et al., 2019; Stern et al., 2018; and González et al., 2019) are of very high quality (CCAT 37–38).

Stern et al. (2018) examined the longitudinal association between CM (childhood victimization: physical abuse and neglect, sexual abuse, emotional abuse and neglect, bullying by peers, domestic violence) and ADHD from childhood into young adulthood. Participants were members of the Environmental Risk (E‐Risk) Longitudinal Twin Study, which tracks a population‐representative birth cohort of 2232 British twins. The findings highlighted comorbidity between ADHD and conduct disorder as an important risk factor for CM, using the twin design to take familial confounding into account.

Boyd et al. (2019) used an Australian population‐based cohort of 3778 mother‐child pairs linked with data from the state child protection agency to study the association between CM (non‐sexual: physical, emotional abuse or neglect, sexual abuse), and attentional problems in adolescence and young adulthood. Also, they explored whether outcomes depended on the type of maltreatment (sexual vs. non‐sexual).

González et al. (2019) used the Boricua Youth Study, a cohort of 2480 individuals of Puerto‐Rican heritage (residing in both the US and Puerto‐Rico), to evaluate the association between increased exposure to multiple forms of CM (physical abuse, emotional abuse, sexual abuse, neglect, and foster placement) on the risk of having persistent ADHD symptoms and diagnosis later.

Calhoun et al. (2019) examined the different impacts of CM (emotional abuse and neglect, physical abuse and neglect, sexual abuse) and harsh parenting as predictive factors for the development of ADHD symptoms among other psychiatric disorders through years.

Hunt et al. (2017) examined the association of exposure to childhood abuse (emotional, physical, sexual), neglect (emotional and physical); and other adverse childhood experiences and whether they have higher odds of receiving an ADHD diagnosis and experiencing externalizing and internalizing problems. They tested and highlighted contributing social factors such as ethnicity.

Guendelman et al. (2016) explored the long term impact of experiencing early CM (physical abuse, sexual abuse and neglect) on girls with ADHD highlighting self‐harm, anxiety, depression, eating disorders and lower well‐being as risk factors.

Wong et al. (2022) study analyzed hospital administrative records of psychiatric diagnoses dating between 2001 and 2019 of 7473 patients aged 0–19, who had experienced childhood maltreatment and compared them to the records of 26,834 patients who had not.

### Heterogeneity

Methodological heterogeneity precluded meta‐analysis (Bender et al., 2018). There was a great variety in research methods across the studies, including with regard to types of design, forms of CM, number and ages of follow‐up assessments, characteristics of participants (e.g., age), definitions and types of maltreatment. For example, 7 studies tested multiple types of CM while 4 studies tested only 1 type of CM (2 deprivation, 1 malnutrition, 1 emotional neglect). In addition, the assessment of ADHD differed greatly across studies (diagnosis, symptoms, traits, behaviors) (see Table S1). This heterogeneity meant that a meaningful and unbiased pooling of results was impossible (Bender et al., 2018).

### The evidence for a causal link between ADHD and CM

We examined whether each study fulfilled the four Bradford Hill criteria that can be applied to individual studies (see Table S2).

#### Strength of association/effect size

We applied Cohen's formula to define effect sizes: small, medium, large (Cohen, 1962) (see Tables S2 and S3). In five studies the effect size was medium or large but only regarding certain associations: between early deprivation and ADHD (Kennedy et al., 2016), and between physical abuse and ADHD diagnosis Hunt et al. (2017). Stern et al. (2018) found a medium to large effect size for victimization (abuse and neglect) and ADHD in childhood and young adulthood. Wong et al. (2022) found a large effect size for the experience of maltreatment and the development of psychiatric symptoms, including ADHD. McLaughlin et al. (2010) found a medium effect size for ADHD symptoms and early deprivation. Four studies found small to medium effect sizes: González et al. (2019), Boyd et al. (2019), Calhoun et al. (2019), and Galler et al. (2012). Two studies, Guendelman et al. (2016) and Young et al. (2011), found small effect sizes.

#### Temporal order (temporality)

Seven studies met criteria for temporal ordering, while four studies did not fulfill the criterion because the CM and ADHD were measured at the same point. Two studies provided evidence that ADHD precedes CM. Using data from early childhood, they indicate that childhood ADHD is associated with a higher risk of various forms of maltreatment occurring later in life (Guendelman et al., 2016; Stern et al., 2018). Although chart review (Guendelman) or repeated parental interviews (Stern) established contemporaneous accounts of CM in these studies, it is still possible that CM could have occurred earlier in life than was reported in charts or by parents” and, regarding Galler et al. (2012), Kennede et al. (2016), McLaughlin et al. (2010), Wong et al. (2022) and Hunt et al. (2017). In these studies it is possible that ADHD symptoms or diagnoses were present prior to the first assessment point in the study–for example, in the Galler study, ADHD symptoms were first measured at age 5 and in the Kennedy study, at age 6. Two studies discussed the possibility of bidirectional causality between ADHD and CM. The design of these studies, however, did not allow for bidirectionality to be formally tested (González et al., 2019; Guendelman et al., 2016) although the Guendelman study did offer evidence that continuing exposure to CM might lead to persistence of ADHD symptomatology across development (Guendelman et al., 2016).

#### Dose response relationship

Four studies met criteria for a dose response relationship between CM and ADHD. Three studies (González et al., 2019; Hunt et al., 2017; Kennedy et al., 2016; Stern et al., 2018) found that cumulative CM experiences (exposure to multiple forms of CM and/or frequently) are associated with more severe ADHD symptoms: Kennedy et al. (2016) found that when exposure to early deprivation was >6 months, maltreatment was associated with more inattention and overactivity symptoms in children and persistent ADHD in adults, than children with <6 months exposure. Hunt et al. (2017) found that children exposed to multiple adverse experiences of physical and emotional neglect and abuse were more likely to have an ADHD diagnosis than those without any adverse childhood experiences. González et al. (2019) found that more experiences of physical and emotional abuse are associated with a higher risk of ADHD diagnosis and/or persistent ADHD symptoms compared to a single experience of abuse or neglect. Stern et al. (2018) found that childhood exposure to moderate CM (physical abuse and neglect, sexual abuse, emotional abuse and neglect) was associated with higher risk of ADHD diagnosis compared to children who were not exposed to maltreatment, and those exposed to severe or multiple forms of CM had even higher risk. None of the studies examined whether having more ADHD symptoms was associated with a higher risk of CM.

#### Plausibility/biological plausibility

Ten of the 11 studies suggested plausible—but not proven—mechanisms for the association between CM and ADHD. There were four predominant plausible explanations:

(a) ADHD with comorbidities is associated with a higher risk of maltreatment: Stern et al. (2018), Calhoun et al. (2019), and Guendelman et al. (2016) proposed that the association between CM and ADHD and CM is largely found among children with comorbid conduct disorder (e.g., conduct disorder), and it is likely that the child's disruptive behaviors might have provoked child maltreatment.
(b) Early maltreatment, deprivation or malnutrition has a negative impact on neurodevelopment: Wong et al. (2022), Kennedy et al. (2016), McLaughlin et al. (2010), and Galler et al. (2012) propose that severe early deprivation or malnutrition can disrupt the development of the brain, causing cognitive deficits and increasing the risk of development of ADHD symptoms.
(c) Confounders could provoke both CM and ADHD: Hunt et al. (2017) and Boyd et al. (2019) propose that factors such socioeconomic status, and parental mental ill health influence both the risk of CM and the risk of an ADHD diagnosis.
(d) Nature‐nurture interplay: González et al. (2019) discuss, without testing it, the possible interplay between environment and genes, where ADHD running in the family might make both parents and children vulnerable for developing ADHD symptoms and could affect parenting practices and children's responses, potentially leading to CM.

Only two studies (Kennedy et al., 2016 and Stern et al., 2018) met the criteria for all four core BH criteria—*strength, dose‐response*, *plausibility, and temporality* (see Table S1). These two studies suggested different temporal orders for the relationship between CM and ADHD and different plausible mechanisms.

## DISCUSSION

This review aimed to examine systematically the published research evidence about the links between CM and ADHD as described in prospective longitudinal studies and to examine evidence for possible causal processes. All 11 studies found an association between CM and ADHD. As regards causality, only two of the highest quality studies (Kennedy et al., 2016 and Stern et al., 2018) met all four of the core Bradford‐Hill criteria and suggested opposite directions of causality and contrasting mechanisms of action for the association between CM and ADHD.

Below, we consider each of the Bradford‐Hill Criteria in the context of the wider literature.

*Strength of the association* between ADHD and CM: In accordance with the previous literature (Brown et al., 2017; Capusan et al., 2016; Ouyang et al., 2008) our systematic review revealed significant associations between CM and ADHD. Both of the studies that met all four core Bradford‐Hill criteria had medium to large (Stern et al., 2018) or large effect sizes (Kennedy et al., 2016).

*Temporality*: Children with ADHD have a higher risk of maltreatment events during childhood and adulthood compared to groups of children with other neurodevelopmental conditions and those with typical development (Christoffersen, 2019; Guendelman et al., 2016; Kennedy et al., 2016; Stern et al., 2018). Conversely, childhood CM experiences: physical abuse and neglect, emotional abuse and neglect, sexual abuse and early deprivation are associated with a higher risk of having ADHD symptoms later in life compared to non‐maltreated children (Boyd et al., 2019; Calhoun et al., 2019; Wong et al., 2022). Additionally, early malnutrition has been marked as a risk factor with a long‐lasting epigenetic dysregulation impact on increasing ADHD symptoms later in life (Galler et al., 2012; Peter et al., 2016).

*Dose‐response*: Our findings about dose‐response are compatible with many longitudinal studies, both retrospective and prospective: exposure to multiple childhood adversities, to poly‐victimization or more frequent maltreatment, during childhood, is associated with increased risk of ADHD symptoms in childhood and adulthood (Björkenstam et al., 2018; Stern et al., 2018; Sugaya et al., 2012). There is evidence in the literature about the cumulative impact of more and/or more severe ADHD symptoms on the risk of neglect and abuse (Ouyang et al., 2008; Vogel et al., 2018), but this was not assessed by the studies we examined in this review.

*Plausibility*: The studies included in our review offer a range of plausible—although largely untested—mechanisms for the relationship between CM and ADHD (see Results, Discussion). What is clear from the review is that there are likely to be underlying biological and environmental factors that might exacerbate or minimize the strength of this relationship and the included studies, despite their highly quality, cannot resolve the conflicts regarding whether any, or all, of these plausible mechanisms are correct.

In order to critically discuss the reviewed papers as a whole, we now examine the five additional Bradford Hill criteria that can be applied across all studies: *coherence, consistency, specificity, analogy* and *experimental evidence* (Howick et al., 2009).

*Coherence:* Although the majority of the studies in the previous literature were retrospective and cross‐sectional, our results are coherent with most of them in that they revealed significant associations between ADHD and CM, which appear to start from childhood and persist in adulthood (Briscoe‐Smith & Hinshaw, 2006; Brown et al., 2017; Capusan et al., 2016; Ouyang et al., 2008). Two included studies suggested ADHD may be a risk factor for children experiencing maltreatment later in life (Guendelman et al., 2016; Stern et al., 2018) reflecting findings in previous literature suggesting (Brown et al., 2017; Christoffersen, 2019). Conversely, seven studies (Boyd et al., 2019; Calhoun et al., 2019; Galler et al., 2012; González et al., 2019; Hunt et al., 2017; Kennedy et al., 2016; Wong et al., 2022; Young et al., 2011), found that CM experiences are associated with a higher risk of having ADHD symptoms later in life compared to non‐maltreated children, reflecting similar findings in the previous literature (Golm et al., 2020; Karatekin et al., 2018; Sonuga‐Barke et al., 2017; Villodas et al., 2015). All of studies included in the review confirmed previous research evidence (Baldwin et al., 2023; Dinkler et al., 2017; Sonuga‐Barke et al., 2017)—that there are both biological and environmental factors that might exacerbate or minimize the risk of experience ADHD and/or CM, and the strength of their relationship.

*Consistency:* Consistent with the studies examined in this review, previous studies of various types have identified a variety of associations between CM and ADHD. All types of maltreatment are not always associated with the same symptoms of ADHD (Sanderud et al., 2016); not all maltreated children will develop ADHD symptoms (Fuller‐Thomson et al., 2014; Sonuga‐Barke et al., 2017), and not all children with ADHD symptoms experience child maltreatment—in fact, the overwhelming majority do not (Ouyang et al., 2008). There is consistency in the finding of the association between CM and ADHD across different study designs for example, retrospective longitudinal (Fuller‐Thomson et al., 2014), behavioral genetic (Capusan et al., 2016). Some study designs are more appropriate for examining causality than others for example, certain types of quasi‐experimental design are particularly well suited to this and use of more appropriate designs will be crucial, in future, to establish stronger inference of causal relationship between ADHD and CM.

*Specificity*: The literature on the relationship between CM and ADHD is non‐specific for the reasons stated in the paragraph above.

*Analogy*: There is some analogy here: child maltreatment is associated with increasing risk of internalizing and externalizing symptoms throughout the lifespan (Sugaya et al., 2012; Wang et al., 2020).

*Experimental Evidence:* The follow‐up of the BEIP randomized controlled trial itself (examining outcomes related to the foster care intervention, which discontinued the severe maltreatment of institutionalization) demonstrated no effect of removing young children to foster care on ADHD symptoms (Humphreys et al., 2015). Clinical Trials on children with ADHD and their parents have presented positive effects of treatments, trainings, or programs on decreasing ADHD symptoms and the risk of harsh parenting (Mah et al., 2021; Sciberras et al., 2019). However, there is no research evidence from trials to support a reduction of the risk of other forms of maltreatment in children with ADHD.

*Limitations to the evidence for causality in the 11 included papers*.

Although all the included studies are longitudinal and prospective, and the overall quality of the reviewed papers is high, there are some limitations that prevent clear conclusions about causality (see Appendix S1):

1. There are issues regarding the timing of CM and ADHD assessment which cannot be overcome in the prospective longitudinal design as discussed above.
2. It is already well known the there is an important role for genes in susceptibility for ADHD (Demontis et al., 2019) and recent studies also highlight genetic susceptibility for CM (He & Li, 2022; Li & Lee, 2012; Warrier et al., 2021). However, in these prospective longitudinal studies, only one study, Stern et al. (2018), examined the potential influence of genetic factors in the association between CM and ADHD.
3. The range of cultural settings where the nature of the relationship between CM and ADHD has been examined is limited. Studies have been mainly in Western samples, so do not fully account for socio‐cultural factors that may shape the association between ADHD and CM. Studies conducted in a wider range of social environments are required to explore generalizability of findings.
4. Traditional linear epidemiological methods with a reliance on specific exposures leading to specific outcomes cannot disentangle the complex relations between CM and ADHD. A more sophisticated analytic research approach, where multiple outputs and inputs can be assessed simultaneously, including controlling for genetic factors alongside environmental factors, are required (Silverman et al., 2021). Other research designs are needed too, such as intergenerational designs in which it is possible to assess the development of the association between parental ADHD and CM in their offspring. Quasi‐experimental studies can also be used to strengthen causal inference (Baldwin et al., 2023), or experimental studies that examine interventions that prevent exposure to CM in relation to onset or worsening of ADHD symptoms. Also, the in the interpretation of the effect sizes and Cohens' *d* in future studies should be done carefully in consideration the research and clinical context in order not to be misleading or inaccurate (Carey et al., 2023).

There are few limitations to consider about the present systematic review too. Our review wasn't register in PROSPERO. We had limited information from the included studies to assess the association between each type of CM separately with ADHD. Our findings should also be interpreted in the context of limitations. First, the interpretation of BH criteria should not be regarded as rigid criteria, but instead as tool to provide serious considerations regarding causality and as a framework for further analyses and studies. For example, it is difficult to understand how the plausibility criteria is applied in this context. Also, confounding is major factor to consider in the context of causal inference, but this was not part of the Bradford criteria. Second, it would have been helpful if it had been possible to examine some of the key theoretical models for the impact of maltreatment, such as the dimensional deprivation versus threat model (McLaughlin et al., 2021), but the wide range of different types of CM and the small number of studies of each precluded this.

## CONCLUSION

The present systematic review reveals an association between CM and ADHD, as well as compelling but not yet conclusive evidence for child maltreatment causing ADHD *and* for ADHD provoking maltreatment. Therefore, further research should be done to detangle this association in order to prevent the risk of maltreatment in children with ADHD and provide better assessment and therapeutic options in children with history of maltreatment to prevent the risk of psychopathology.

## AUTHOR CONTRIBUTIONS

**Paraskevi Bali**: Conceptualization; Data curation; Formal analysis; Investigation; Methodology; Project administration; Software; Writing – original draft; Writing – review & editing. **Edmund Sonuga‐Barke**: Conceptualization; Methodology; Supervision; Writing – review & editing. **Christina Mohr‐Jensen**: Conceptualization; Methodology; Project administration; Supervision; Writing – review & editing. **Ditte Demontis**: Supervision. **Helen Minnis**: Conceptualization; Methodology; Project administration; Supervision; Writing – review & editing.

## CONFLICT OF INTEREST STATEMENT

Ditte Demontis serves on the JCPP Advances Editorial Advisory Board. The remaining authors have declared they have no competing or potential conflicts of interest.

## ETHICAL CONSIDERATIONS

No ethical approval was required for this research review.

## Supporting information

Supporting Information S1

## Data Availability

The data that support the findings of this study are available on request from the corresponding author.
