# Supplementary material for: Is there evidence of a causal link between childhood maltreatment and attention deficit/hyperactivity disorder? A systematic review of prospective longitudinal studies using the Bradford‐Hill criteria
Source: JCPP Adv. 2023 May 27;3(4):e12169. doi: 10.1002/jcv2.12169 (PMC10694545; doi:10.1002/jcv2.12169)
Supplement: Supplementary file 1 — Supporting Information S1 [file JCV2-3-e12169-s001.docx]

**Supporting Information**

**Table S1-** Detailed Description of the studies

| **Studies** | **Country** | **Sample** | **Number of follow-ups after the first assessment** | **Number and types of participants** | **Gender** | **Exposure** | **Outcomes** | **Type of ADHD** | **CCAT**  **(max=40)** |
| --- | --- | --- | --- | --- | --- | --- | --- | --- | --- |
| *Studies investigating early deprivation* | | | | | | | | | |
| McLaughlin et al., 2010  (BEIP) | Romania | Bucharest Early Intervention Study | 3 follow-ups | 117 children raised in institutions  49 children community control | Male and Female | Early deprivation | ADHD, anxiety, depression, and disruptive behaviour disorders in childhood | ADHD diagnosis & symptoms: Inattention, Hyperactivity, Impulsivity  (DSM-IV) | 38 |
| Kennedy et al., 2016  (ERA study) | UK | The English and Romanian Adoptees project | 2 follow-up | Children and young adults: 165 Romanian adoptees &  52 UK adoptees | Male and Female | Early deprivation | ADHD, ASD, Disinhibited social engagement, anxiety, major depression, conduct disorder | ADHD symptoms  (DSM-5) | 36 |
| *Malnutrition* | | | | | | | | | |
| Galler 2012 | USA | Barbados nutrition study | 2 follow-ups | 269 participants: 124 children & 145 adults | Male and Female | Infant malnutrition | ADHD symptoms in childhood and adolescence | ADHD symptoms: Inattention, Hyperactivity  (DSM-IV) | 30 |
| *Emotional Neglect* | | | | | | | | | |
| Young et al., 2011 | UK | Longitudinal school-base survey | 2 follow-ups | 1.700 children & adolescents | Male and Female | Parental neglect & control | ADHD, anxiety, conduct disorder, depression in adolescence | ADHD diagnosis and symptoms  (DSM-IV) | 35 |
| **Table S1 –** Detailed Description of the studies**,** continued | | | | | | | | | |
| *Multiple forms of abuse and neglect* | | | | | | | | | |
| Wong et al., 2022 | China | Longitudinal hospital based administrative records | Multiple | 7473 children with CM experience  26,834 control with no CM | Male and Female | Non-sexual CM: physical abuse, emotional abuse, and neglect  Sexual abuse | ADHD,  psychosis depression  bipolar disorder, anxiety, conduct disorder/oppositional defiant disorder  personality disorders suicide and self-inflicted injury | ADHD diagnosis (ICD-9) | 33 |
| Stern et al., 2018 | UK | Environmental Risk (E-Risk) Longitudinal Twin Study | 3 follow-ups | 2.232 children | Male and Female | Childhood abuse/ neglect  Childhood ADHD | ADHD in childhood and young adulthood  Abuse and neglect in young adulthood | ADHD diagnosis  (DSM-IV) | 38 |
| Gonzalez et al., 2019 | USA | Boricua Youth Study (BYS) | 3 follow-ups | 2.480 children & adolescents | Male and Female | Maltreatments & abuse  Emotional abuse | ADHD diagnosis in childhood and adolescence  Emotional abuse, physical abuse, and foster placement | ADHD diagnosis  (DSM-IV) | 38 |
| Boyd et al., 2019 | Australia | The Mater-University Study of Pregnancy (MUSP) | 2 follow-ups | 3.778 pairs: mother-child | Male and Female | Childhood maltreatment  neglect, emotional and physical abuse in childhood | Attentional problems in adolescence and young adulthood | ADHD diagnosis  (DSM-IV) | 37 |
| Calhoun, 2019 | USA | Longitudinal Study of Marijuana Use and Neurodevelopment | 2 follow-ups | 529 early adolescent youth | Male and Female | Childhood maltreatment & harsh parenting | ADHD symptoms general anxiety disorder and depression, conduct disorder | ADHD diagnosis (DSM-IV) | 35 |
| Hunt et al.,2017 | USA | The Fragile Families and Child Wellbeing Study (FFCW) | 4 follow-ups | 4.898 children | Male and Female | Childhood maltreatment | ADHD, externalising and internalising problems in childhood | ADHD diagnosis  (DSM-IV) | 35 |
| Guendelman et al., 2016 | USA | The Berkeley Girls with ADHD Longitudinal Study (BGALS) | 2 follow-ups | 140 girls: children & adolescents | Female | Childhood ADHD | ADHD symptoms, suicide attempts, anxiety, depression, eating disorders in young adulthood | ADHD diagnosis  (DSM-IV) | 36 |

**Table S2** – core Bradford- Hill criteria for the individual studies

|  | **Effect size: Was there a moderate or large effect not attributable to confounding?** | **Temporal sequence** | **Dose- responsiveness and reversibility** | **Plausibility** |
| --- | --- | --- | --- | --- |
| **Wong et al., 2022** | **Yes:**  CM was sig. associated with ADHD: HR: 10.62 (95% CI: 8.35- 13.50), p<0.001 | **Yes:** the study followed participants (aged 0-19) for ten years assessing the CM and ADHD from early childhood and conducting multiple follow-ups for ten years.  They included only participants with no psychiatric record before the CM events. | **No**: Not assessed in study | **Yes**: The gender and socioeconomic status of the families could influence the association between CM and ADHD.  The structural and functional brain changes are associated with symptoms of psychiatric disorders. |
| **Kennedy, et al.,**  **(ERA study)** | **YES:** Strong association between deprivation and individuals meeting ADHD thresholds in adolescence and early adulthood. ADHD in Romanian children>6months deprivation:  Adolescence: F (1, 136) = 30.32, p < 0.001,  Early adulthood: F (1, 135) = 21.05, p < 0.001 | **YES:** the study assessed the ADHD and CM from early childhood and multiple follow-ups until adulthood | **Yes:** Children who were exposed for a longer period to a deprivation (>6 months) had higher rates of Inattention and overactivity symptoms. | **YES**: Severe deprivation disrupted the development of the brain causing cognitive issues and increasing the risk of development of ADHD symptoms that couldn’t be explained by other factors. |
| **Gonzalez 2019** | **YES**: Emotional abuse 2.4 [1,45-4,03] p< 0.001 with medium effect size. | **No**: ADHD was assessed at least one time during the ages 5-13. No information about onset of either ADHD symptoms or CM or their temporal ordering. | **YES:** more physical and emotional abuse higher risk of having ADHD diagnosis and persistent ADHD symptoms by 40% [OR 1.40 (1.08, 1.81), p = 0.01]. | **YES**: The possible interplay between environment and genes could explain the association between CM and ADHD |
| **Boyd et al., et al. (2019)** | **No:** at the age 21-year assessment, a history of non-sexual CM was associated with ADHD symptoms (B=0.33 (SE=0-17), p=0.044) but with small effect size. | **No**: ADHD symptoms were measured at 14 and 21 years. Child maltreatment was measured before the age of 14. No knowledge about whether the ADHD began prior to the CM or vice versa. | **No**: Not assessed in study | **YES**: Socioeconomic factors affect the development of ADHD and the risk of experiencing maltreatment as well as the strength of their links. |
| **Calhoun et al., 2019** | **YES**: CM was a stronger predictor for ADHD only in wave 1: (IRR) 2.23 [1.78, 2.81], p<0.001. and not in the wave 2: 1.01 [.77, 1.34]. | **No**: The assessment of CM and ADHD started at the age of 10, so symptoms could already pre-exist in children before that time. | **No:** Not assessed in study. | **Yes:** The comorbidity of ADHD with conduct disorder and the pre-existing mental health symptoms are plausible explanations for the development of the association between CM and ADHD. |
| **Stern et al., 2018** | **Yes**: Childhood ADHD was sig. associated with abuse/neglect up to age 18 years (B=0.13, p=0.011). Robust to adjustment for IQ at age 5 and parental SE (B=0.14, p=0.013). | **Yes:** CM and ADHD were assessed repeatedly when the children were 5, 7, 10, and 12 years of age. | **Yes:**  *Children*: moderate abuse/neglect=  2.02 odds for ADHD  severe abuse/neglect= 2.78 odds for ADHD  *Young adults:* moderate abuse/ neglect during adolescence= 2.76 odds for ADHD.  severe abuse/neglect in adolescence = 3.86 odds for ADHD | **Yes:** The association between ADHD and CM is largely found among children with comorbid conduct disorder |
| **Hunt et al.,2017** | **Yes**: Physical neglect and ADHD: B= 0.071 SE=0.07; 1.19 [0.79,1.78].  Physical abuse and ADHD: B=0.24 SE=0.06; 1.63 [1.17,2.28], p<.01.  Emotional abuse and ADHD: B= 0.18 SE=0.05; 0.97 [0.72,1.31], P <0.001. Emotional neglect and ADHD: B=0.17 SE= 0.04; 1.19 [0.91,1.57], P<0.001. Domestic violence B= and ADHD: 0.16 SE=0.04; 0.84 [0.64,1.10], P<0.05. | **No**: Although there were various follow-ups from age 1, CM was assessed with follow-ups at 1, 3, 5, and 9 years lifetime ADHD was only assessed at age 9 and symptoms could already pre-exist in children before that assessment. | **Yes**: children with 0 ACEs, those with 2, 3, and 4 or more ACEs had 1.7 (p<.01), 1.8 (p<.01), and 2.7 (p<.001) times the odds, respectively, of having an ADHD diagnosis. | **Yes:** The different indicators of socioeconomic wellbeing, ethnicity and parental mental health influence the risk of CM and the development of internalizing and externalizing symptoms and ADHD diagnosis. The experience of multiple forms of CM and additional ACEs increase the risk of receiving ADHD diagnosis |
| **Guendelman et al., 2016** | **Yes**: Participants with ADHD were more likely to have experienced maltreatment relative to comparisons, χ2 (1, N = 228) = 4.75, p = 0.02. adjusting for demographic, prenatal. | **Yes**: Study evaluated participants in childhood, adolescence, and young adulthood. ADHD pre-existed and was assessed before any onset of CM problems could occur. | **No**: Not assessed in study | **Yes:** The comorbidity of ADHD with self-harm behaviours, eating disorders and internalizing symptoms in girls and women with ADHD could affect the risk the strength of the links with CM |
| **Galler et al. (2012)** | **Yes**: ADHD symptom scores at age 5-18 associated with infant malnutrition (B=0.09, SE=0.03, p<0.01) with medium effect size compared to healthy controls. Effect was non-significant for symptoms of hyperactivity/impulsivity but was driven by differences related to symptoms of inattention. | **Yes**: infant malnutrition was assessed from the first year of life of children. Both malnutrition and ADHD symptoms were evaluated at three time points during the ages of 5-11 and only ADHD at 37-43 years. | **No**: Not assessed in study | **Yes:** The infant malnutrition might cause cognitive impairments such ad executive function and attentional issues and ADHD diagnosis |
| **Young et al., 2011** | **No**: between parental behaviours as perceived during childhood and ADHD: B=0.26 (0.14–.39) p <.001 and for perceived neglect of children and ADHD: B=0.30 (0.00–0.59) p= 0.050 but effect sizes small and could have been confounded by factors affecting child’s perception. | **No**: the study assessed ADHD and emotional maltreatment after the age of 11, so, we have no knowledge about the onset of either ADHD symptoms or CM or their temporal ordering. | **No**: Not assessed in study | **Yes**: Family structure, household socioeconomic status and the possible pre-existing psychiatric disorder in children may affect the association between children’s perception of parental neglect and psychopathology. |
| **McLaughlin et al., 2010**  **(BEIP)** | **Yes:** Children who were institutionalized had a greater number of ADHD symptoms of inattention [F(1,147) = 21.3, p < .001]. Hyperactivity [F(1,147) = 11.4, p <.001], and impulsivity [F(1,147) = 16.6, p<.001] compared to community controls. | **Yes:** The study assessed both ADHD symptoms and CM and There were various follow-ups from 30 and 42 months. | **No**: Not assessed in study | **Yes:** Severe deprivation disrupted the development of the brain causing cognitive issues and increasing the risk of development of ADHD symptoms that couldn’t be explained by other factors. |

**Table S3** – Details of effect sizes- Cohen’s d score

| **Cohen’s d score**  **Size of effect** – Cohen’s d score  Small= 0.20 – 0.40  Medium= 0.50- 0.70  Large= >0.80 | |
| --- | --- |
| **Wong et al., 2022** | **Yes:**  CM was sig. associated with ADHD: 1.30 |
| **Kennedy et al., 2019** | **Large**  Symptoms of ADHD in the non-deprived UK and Romanian adoptees exposed to institutional deprivation:  Adolescence: Cohen’s d =0.86  Young adulthood: Cohen’s d =0.83 |
| **Gonzalez 2019** | **Small to Medium**  Emotional abuse- ADHD Cohen's d= 0.51  Physical abuse- ADHD Cohen's d=0.296  Sexual abuse- ADHD Cohen's d= 0.037  Neglect-ADHD Cohen's d= 0.22 |
| **Boyd et al., et al. 2019** | **Small to Medium**  Non-sexual abuse: - Attention problems (parent report CBCL age 14) Cohen's d =0.44  - Attention problems (youth report YSR age 14) Cohen's d =0.322  - Attention problems (youth report YASR age 21) Cohen's d =0.15  Sexual abuse:- Attention problems (parent report CBCL age 14) Cohen's d =0.21  -Attention problems (youth report YSR age 14) Cohen's d =0.19  - Attention problems (youth report YASR age 21) Cohen's d =0.03 |
| **Calhoun et al., 2019** | **Small to medium**  Childhood trauma- ADHD  Wave 1: Cohen’s d=0.44  Wave 2: Cohen’s d=0.005 |
| **Stern et al., 2018** | Medium to large*   \| Associations between victimization and ADHD in childhood:  Moderate neglect/abuse Cohen's d= 0.38  Severe neglect/abuse Cohen's d= 0.56  Any Cohen's d= 0.309  Domestic violence Cohen’s d=0.46  Exposure to one type of victimization Cohen’s d=0.43  Exposure to two or more type of victimization Cohen’s d=0.76 \| Associations between victimization in adolescence and ADHD in young adulthood.  Moderate neglect/abuse Cohen's d= 0.56  Severe neglect/abuse Cohen's d= 0.74  Domestic violence Cohen’s d=0.45  Exposure to one type of victimization Cohen’s d=0.37  Exposure to two or more type of victimization Cohen’s d=0.658 \| \| --- \| --- \|   **There are slight differences from the effect sizes cited in the Stern paper due to differences in accounting for covariates* |
| **Hunt et al.,2017** | Only Physical abuse – ADHD has a **large effect**  Physical neglect (past 12 months) - Parent-reported ADHD diagnosis at age 9-year assessment Cohen's d= 0.096  Physical abuse (past 12 months) -Parent-reported ADHD diagnosis at age 9-year assessment  Cohen's d= 0.26  Emotional abuse (past 12 months)- Parent-reported ADHD diagnosis at age 9-year assessment Cohen's d= -0.0  Emotional neglect (past 12 months) -Parent-reported ADHD diagnosis at age 9-year assessment Cohen's d= 0.09 |
| **Guendelman et al., 2016** | **Small**  ADHD-related symptoms  Mother CBCL attention problem T score Maltreated VS Non-Maltreated: Cohen’s d=0.38  Mother SNAP-IV inattention (0-9) Maltreated VS Non-Maltreated: Cohen’s d=0.4  Mother SNAP-IV HI (0-9) Maltreated VS Non-Maltreated: Cohen’s d=0.44  Teacher SNAP-IV inattention (0-9) Maltreated VS Non-Maltreated: Cohen’s d=0.03  Teacher SNAP-IV HI (0-9) Maltreated VS Non-Maltreated: Cohen’s d=0.28 |
| **Galler et al. (2012)** | **Small to Medium**  Childhood malnutrition- Inattention (adult self-report CAARS) Cohen's d = 0.63  Childhood malnutrition- Hyperactivity (adult self-report CAARS) Cohen's d = 0  Childhood malnutrition- DSM-IV ADHD symptoms (adult self-report CAARS) Cohen's d = 0.63  Childhood malnutrition- ADHD Index (adult self-report CAARS) Cohen's d = 0.63 |
| **Young et al., 2011** | **Small**  ADHD association with perceived parenting  ADHD- typical parenting Cohen's d = 0.186  ADHD- Moderate parenting Cohen's d =0.14  ADHD-Neglectful & controlling parenting Cohen's d =0.4 |
| **McLaughlin et al., 2010** | **Medium**  ADHD – children reared in institutions Vs community controls Cohen’s d= 0.69  Inattention- institution Vs community Cohen’s d=0.67  Hyperactivity- institution Vs community Cohen’s d=0.49  Impulsivity- institution Vs community Cohen’s d=0.59 |

**Appendix S1 - Problems with establishing temporality within individual studies.**

*Limitations to the evidence for causality*

Although all the included studies are longitudinal and prospective, and the overall quality of the reviewed papers is high, there are issues regarding the timing of CM and ADHD assessment which cannot be overcome in the prospective longitudinal design (see Supplementary Information). For example, there is no assessment of ADHD before the age of 9 in Hunt’s study (2017). Both the Young (2012) and Boyd (2019) studies recruited and followed children only after the age of 11 and 14, therefore symptoms could already have pre-existed in children before that assessment (Ouyang, 2008). Galler (2012) found that infant malnutrition preceded cognitive impairments and attentional issues and ADHD diagnosis in adulthood. However, an alternative explanation is that ADHD symptoms in children might have existed before the malnutrition and that could result in the child being more difficult to feed (Schneider, 2019). Also, the Galler (2012) study did not assess additional factors such as parental psychopathology and their possible moderation of the association between malnutrition and ADHD symptoms.

Wong (2022), Boyd (2019), Guendelman (2016), Calhoun (2019) and Gonzalez (2019) simultaneously assessed the association between physical abuse and neglect, emotional abuse and neglect, and sexual abuse (Gonzalez did not assess emotional neglect and abuse) on ADHD symptoms and provided evidence of the long-term association between CM and ADHD, but they do not provide cogent evidence for causality. Although, Wong (2022) is a ten years prospective study with a detailed assessments of CM and ADHD the authors didn’t test the impact of genetic and parental psychopathology. There is discrepancy between parental and self-reports in the Boyd (2019) study: there is a strong association between sexual maltreatment and attentional problems only in self-reports but not in the carers’ reports. Low levels of agreement may be due to systematic differences in the sensitivity of the measures and the methodology of researchers increasing the risk of reporting bias and might affect the quality of outcomes of the study (Baldwin, 2019). The Guendelman (2016) study tried to explain the relationship between ADHD and CM suggesting potential bidirectional links and common risk factors. They tested various socioeconomic and familial factors, but not biological factors. Also, this study was focused only on girls and women, so it is not a representative sample of the population. The Calhoun (2019) study demonstrated that CM predicts ADHD symptoms, among other psychiatric conditions but only in the first wave of the assessment. The authors did not test possible influences of moderator factors such as genetics, parental psychopathology, or socioeconomic situation. The Gonzalez (2019) study didn’t present evidence for temporality and causality between ADHD and CM. The associations between CM forms and ADHD was significant after the authors adjusted for age, sex, income, household education, any parental psychopathology, ADHD medication and comorbidity with PTSD and CD and only physical abuse was significantly associated with ADHD.
